# Pseudosarcomatous Proliferation of Cx43- and Kit-Expressing Interstitial Cell in the Urinary Bladder

**DOI:** 10.4061/2010/961325

**Published:** 2010-12-22

**Authors:** Tamotsu Takeuchi, Masanobu Tanimura, Tsutomu Shimamoto, Masaharu Yasuda, Mutsuo Furihata

**Affiliations:** ^1^Department of Pathology, Kochi Medical School, Kochi 783-8505, Japan; ^2^Division of Urology, Chikamori Hospital, Kochi 780-8522, Japan; ^3^Department of Urology, Kochi Prefectural Aki Hospital, Kochi 784-0027, Japan; ^4^Department of Urology, Kochi Medical School, Kochi 783-8505, Japan

## Abstract

The authors report a case showing proliferation of KIT- and connexin 43-expressing mesenchymal cells of the urinary bladder. A 75-year-old woman had an ulcerated endophytic mass (size, approximately 2 × 2 cm) in the left posterolateral wall. She underwent transurethral resection and subsequent partial cystectomy. The suburothelial mass extended to the muscularis propria. The histopathological analysis revealed spindle-shaped mesenchymal cells that were loosely arranged with myxoid stroma and showed a focal compact fascicular arrangement. In the immunohistochemical analysis, these spindle cells were stained with specific antibodies to KIT and connexin 43. The patient is currently free of disease at 5 years after operation. The proliferating spindle cells in the present case might represent a phenotype of interstitial cells of the lamina propria.

## 1. Introduction

Although proliferation of various nonepithelial cells can occur in the urinary bladder, this phenomenon is rather rare. However, a fundamental understanding of the proliferation of these cells is important to determine the appropriate treatment approach for patients presenting with this phenomenon.

Interstitial cells of the urinary bladder belong to a group of nonepithelial mesenchymal cells that exhibit elongated or satellite-shaped cell bodies and express KIT [1]. Suburothelial ICCs are characterized by the expression of both KIT and a gap-junction protein, connexin 43 (Cx43), to form an interconnection with neighboring interstitial cells [2]. Piotrowska et al. reported that the ICCs were absent in the urinary bladder of patients with megacystis-microcolon-intestinal peristalsis syndrome, which is characterized by a distended unobstructed urinary bladder [3]. Roosen et al. also showed that the cell number of the Cx43-expressing ICCs was significantly increased in the urinary bladder of patients with detrusor overactivity [4]. These findings may support the idea that ICCs act as a pacemaker or a neurotransmitter in the urinary bladder.

KIT expression is clinical important because of the existence of a compound, imatinib mesylate that specifically inhibits tyrosine kinase receptors [5]. Therefore, it is important to know whether KIT-expressing ICCs could be a source for tumorous or pseudosarcomatous proliferation in the urinary bladder. 

Here, we report a case of proliferation of suburothelial KIT- and Cx43-expressing mesenchymal spindle cells in an adult urinary bladder.

## 2. Case Report

**Figure 1:**
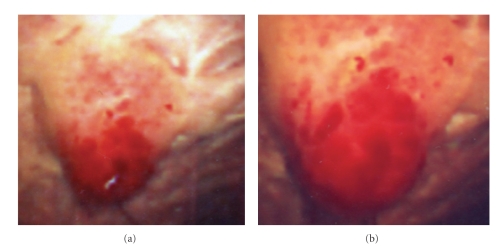
(a) and (b) Cystoscopic appearance of the present mass before transurethral resection.

The patient was a 75-year-old woman with diabetes mellitus and hypertension, but without any history of instrumental treatment. She presented with lower abdominal pain that had persisted for 2 months. Macroscopic hematuria or lower urinary symptoms were not associated. The clinical examinations, including ultrasonography, computed tomography (CT) imaging, and cystoscopy, indicated that the patient had a mass in the left lateral wall of the urinary bladder (Figure 1). CT imaging indicated that the size of mass was approximately 1.5 × 1.5 cm at the initial diagnosis. She underwent transurethral resection; however, the residual mass remained after transurethral resection. Notably, CT imaging, just prior to transurethral resection, showed that the size of the mass increased to 2.0 × 2.0 cm within 1 month. Pathological examination of the resected tumor could not exclude the possibility of leiomyosarcoma. The increase of the mass in a month's time also indicated an aggressive tumor. Ten days after the first transurethral resection, the patient underwent partial cystectomy. The surgical margin was less than 2 mm; however, the patient did not wish to undergo any additional treatments. The patient has been undergoing careful followup examinations including cystoscopy and biopsies. No local recurrence or metastasis has been found 5 years after the patient underwent partial cystectomy. Informed consent was obtained from patient.

## 3. Pathology Findings

**Figure 2:**
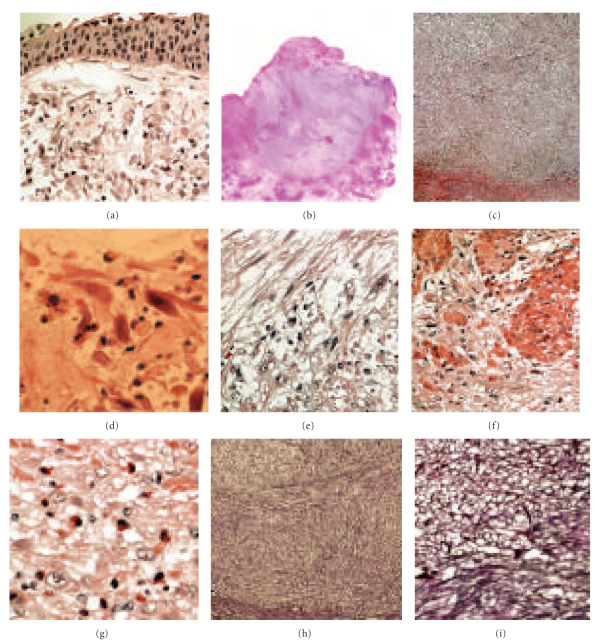
(a) Irregular spindle-to-oval-shaped cells were loosely arranged under the urothelial epithelium (transurethral resection (TUR) specimens). Note the myxoid stroma with a few inflammatory cells. (b) The shape of the mass obtained by partial cystectomy. The upper region of the mass was already resected by TUR. The dome-shaped mass extended from the lamina propria to the deep muscular layer. (c) The mass exhibited a loose and compact arrangement. (d) Spindle-shaped myofibroblastic cells were loosely arranged in the myxoid stroma. Asterisk indicates the mitotic figure. (e) Relatively compact fascicular arrangements of the spindle cells were found in the deep region of the mass. (f) Spindle-to-round-shaped cells invaded the muscle layer. (g) Eosinophil infiltration in the deep region of the mass. (h) and (i) Silver reticulum stain demonstrates reticulin fibers wrapped around individual spindle cells and emphasizes a fascicular growth pattern.

Histopathological examination of the resected tissue specimens showed that loosely arranged spindle-shaped cells proliferated in the myxoid stroma without any significant epithelial cell proliferation. At least partially, these spindle cells showed nuclear atypia accompanied by a few mitotic figures. The spindle cells also showed focal fascicular arrangement. The representative findings are shown in Figure 2.

We observed loosely arranged spindle-shaped cells proliferated in the lamina propria; these cells extended to the deep muscle propria in a dome-shaped formation and accompanied the inflamed myxoid stroma (Figure 2). Various inflammatory cells, most of which were eosinophils and lymphocytes, were found in the myxoid stroma. Some spindle cells exhibited “cigar-shaped” nuclei, which are often found in leiomyosarcoma; further, fascicular arrangement of the spindle cells, which is also often found in leiomyosarcoma, was observed. The number of mitotic structures was approximately 2–4/10 high-power fields. Nuclear atypia was found in many proliferating spindle cells.

**Figure 3:**
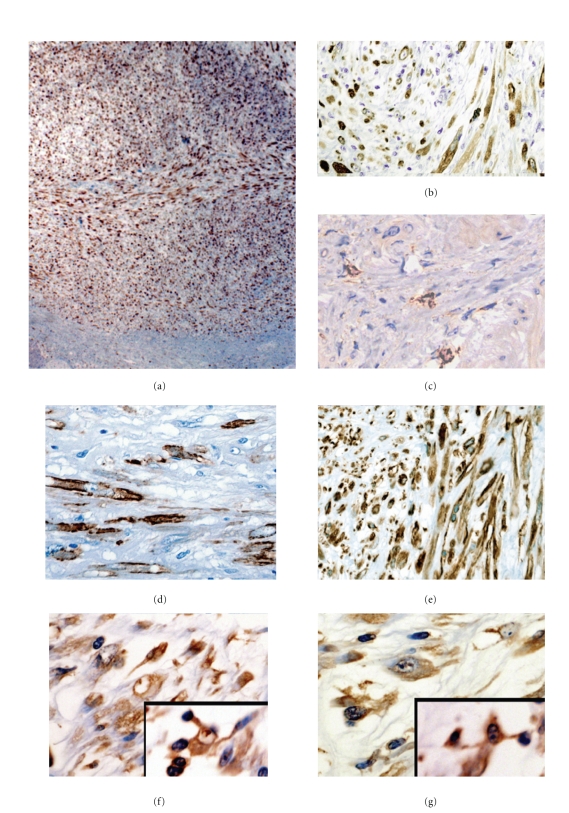
Representative immunohistochemical staining. The proliferating spindle-to-oval-shaped cells showed immunostaining for a specific antibody to KIT ((a) and (b)) with an intensity similar to that found in interstitial cells outside the mass (c). The proliferating cells also stained for *α*-smooth muscle actin (d) and vimentin (e). Note the staining with a specific antibody to connexin 43 (Cx43) (f) and (g). The inserted figures represent the possible interconnections formed by the projections of the cytoplasm.

Immunohistochemical staining was performed as previously reported [6]. In brief, staining was performed using an automated immunostainer (Ventana; Tuscon, AZ). The representative immunohistochemical stains are shown in Figure 3. In the immunohistochemical analysis, most of the proliferating spindle cells (Figures 3(a) and 3(b)) as well as intact interstitial cells outside the mass (Figure 3(c)) stained positive for KIT (CD117) (Ventana). In addition, the spindle cells showed positive staining for *α*-smooth muscle actin (DAKO, Carpenteria, CA) (Figure 3(d)) and vimentin (DAKO) (Figure 3(e)). Notably, almost all proliferating spindle cells showed strong immunoreactivities to the anti-Cx43 antibodies (Abcam, Cambridge, UK) at the projections of the cytoplasm (Figures 3(f) and  3(g)). In contrast, no significant immunoreactivity was detected with antianaplastic lymphoma kinase (ALK) antibody, anticytokeratin antibodies AE1/AE3 (Boehringer-Mannheim, Indianapolis, IN), CAM5.2 (Becton Dickinson, San Jose, CA), cytokeratin 5/6 (DAKO), desmin (DAKO), h-caldesmon (DAKO), HMB-45 (DAKO), or anti-S-100 (DAKO). 

The final pathological diagnosis varied among pathologists, including 4 consultants. Some pathologists diagnosed the present case as an inflammatory myofibroblastic tumor (IMT). Others believed that the present case should be diagnosed as a low-grade myxoid leiomyosarcoma.

## 4. Discussion

Here, we report a case of proliferation of mesenchymal spindle cells with a unique immunophenotype, that is, KIT and Cx43 expression.

KIT is well characterized as a receptor tyrosine kinase for stem cell factors [7]. The clinical importance of KIT expression is based on the successful treatment with a compound—imatinib mesylate—that specifically inhibits tyrosine kinase receptors [5]. Thus, it is important to recognize the presence of a KIT-expressing tumor to provide the optimum therapy for patients.

In the gastrointestinal tract, KIT-expressing ICCs are believed to act as pacemaker cells that generate spontaneous electrical slow waves and mediate inputs from motor neurons [8]. The findings of another study also indicate that KIT-expressing myofibroblast cells, generally referred to as ICCs, exist in the human urinary bladder [1, 9]. The human urinary bladder contains 2 major types of ICCs, namely, suburothelial ICCs and detrusor ICCs. Suburothelial ICCs and lamina propria-ICCs form a network with neighboring ICCs via the gap-junction protein Cx43. The expression of Cx43 is recognized as a distinct feature of suburothelial ICCs [2]. As demonstrated in Figures 3(f) and 3(g), the spindle cells also showed a cytoplasmic projection that connected them with other spindle cells. Therefore, we think that the proliferating spindle cells in the present case may have the immunophenotype of suburothelial ICCs.

Histopathologically, the present case may have features overlapping those of 4 well-established tumors or tumorous conditions. First, the general morphology somewhat resembled that of an IMT [10]. IMT of the urinary bladder is very rare; however, myxoid stroma with inflammatory change is often found in IMT of the urinary bladder [10]. Recently, a case of IMT, which represented the exophytic tumors arising from the dome of the bladder, in a patient with Von Recklinghausen's syndrome was reported [11].

Loosely arranged proliferating myofibroblastic cells are also observed in IMT. However, the myofibroblastic cells in the present case exhibited nuclear atypia, which may not be accepted as a characteristic of classical IMT by many pathologists. Inflammatory cells of the IMT are usually plasma cells or lymphocytes [10]. In contrast, eosinophils were predominant in the present case. Mitosis was reported to be minimal in IMT; however, we could see mitotic structures in the present case (the number of mitotic structures was approximately 2–4/10 high-power fields). IMT is also characterized by delicate capillaries in the inflamed myxoid stroma. However, the present mass lacked delicate capillary networks. The result of the immunohistochemical study was also not compatible with that of typical IMT. ALK expression has been noted in half of the cases of IMT of the urinary bladder [10]; however, we could not detect any significant ALK expression in the present case. KIT expression is also unusual for IMT [10].

Leiomyosarcoma could be considered in the differential diagnosis of the present case. The fascicular arrangement in the present case may be found in leiomyosarcoma. Myxoid change is also frequently found in leiomyosarcoma of the urinary bladder [12, 13]. However, the cellularity of leiomyosarcoma is more uniform than that observed in the present case. Further, the immunophenotype of the present case, that is, KIT-positive, desmin- or h-caldesmon-negative, was not consistent with that of leiomyosarcoma [13].

The concept of extragastrointestinal stromal tumors has been suggested for various tissues. A case of an extragastrointestinal KIT-positive stromal tumor in the urinary bladder has been reported [14]. However, this tumor was found in the serosa of the urinary bladder and projected to the peritoneal cavity. By contrast, the primary location of the mass in the present case was in the bladder wall. Myxoid stroma, or fascicular arrangement of the proliferating cells, is unusual in extragastrointestinal KIT-positive tumors, which closely resemble gastrointestinal stromal tumors. Compact proliferating cells, which are often found in extragastrointestinal KIT-positive tumors, were not noted in the present case.

Finally, so-called pseudosarcomatous fibroepithelial stromal polyp could be the differential diagnosis of the present lesion. Atypical stromal cells of the pseudosarcomatous fibroepithelial stromal polyp formed short, intersecting fascicles mimicking a smooth muscle tumor like the present lesion. A series of pseudosarcomatous fibroepithelial stromal polyp of the lower female genital tract was reported [15]. Although fibroepithelial stromal polyp occur most commonly in the vagina, similar lesions have also been described in the urinary bladder [16]. However, the present lesion had no proliferating epithelial component, which characterized the fibroepithelial stromal polyp. It may be interesting to examine KIT and Cx43 expression in the proliferating atypical stromal cells of pseudosarcomatous fibroepithelial stromal polyp.

To the best of our knowledge, this is the first report which describes the proliferation of KIT- and Cx43-expressing cells in the urinary bladder. Although, we could not conclude whether the mass in our case represents a tumor with indolent proliferation and favorable outcome or pseudosarcomatous mesenchymal cell proliferation, further case studies might draw a definite conclusion.
